# Identifying subphenotypes of patients undergoing post‐operative delirium assessment

**DOI:** 10.1002/alz.70516

**Published:** 2025-07-16

**Authors:** Emily Margaret Louise Bowman, Daniel F. McAuley, Bernadette McGuinness, Anthony P. Passmore, David Beverland, Henrik Zetterberg, Jonathan M. Schott, Amanda Heslegrave, Elena Veleva, Rhiannon Laban, Aoife Sweeney, Emma L. Cunningham

**Affiliations:** ^1^ Centre for Public Health, Institute of Clinical Science, Royal Victoria Hospital Queen's University Belfast Belfast UK; ^2^ Wellcome‐Wolfson Institute for Experimental Medicine Queen's University Belfast Belfast UK; ^3^ Department of Psychiatry, Warneford Hospital University of Oxford Oxford UK; ^4^ Outcomes Assessment Unit Musgrave Park Hospital Belfast UK; ^5^ UK Dementia Research Institute University College London London UK; ^6^ Department of Neurodegenerative Disease, National Hospital for Neurology and Neurosurgery, Institute of Neurology University College London London UK; ^7^ Clinical Neurochemistry Laboratory Sahlgrenska University Hospital Mölndal Sweden; ^8^ Department of Psychiatry and Neurochemistry Institute of Neuroscience and Physiology the Sahlgrenska Academy at the University of Gothenburg Mölndal Sweden; ^9^ Hong Kong Center for Neurodegenerative Diseases Hong Kong China; ^10^ Wisconsin Alzheimer's Disease Research Center, University of Wisconsin School of Medicine and Public Health University of Wisconsin–Madison Madison Wisconsin USA; ^11^ Dementia Research Centre, Department of Neurodegenerative Disease, National Hospital for Neurology and Neurosurgery, Institute of Neurology University College London London UK

**Keywords:** altered consciousness, biomarkers, cognition, cognitive change, delirium, endotypes, glial fibrillary acidic protein, inattention, latent class analysis, machine learning, neurofilament light chain, phenotyping, post‐operative, post‐operative delirium, subphenotypes, unsupervised clustering

## Abstract

**INTRODUCTION:**

Delirium has heterogeneous etiologies and clinical presentations and is often associated with poor outcomes. Its pathophysiological mechanisms remain largely hypothetical and without targeted pharmacological treatment. This work investigates subphenotypes of patients undergoing delirium assessment based on clinical features and fluid biomarkers.

**METHODS:**

We performed latent class analysis of an observational cohort of older adults undergoing elective surgery.

**RESULTS:**

Two classes were identified, both containing individuals experiencing delirium symptoms, with a higher number in Class 1 (*p* < 0.001). Class 1 were older, less educated, and had more depression (*p* < 0.001). They performed worse in all pre‐operative cognitive assessments (*p* < 0.001) and had more markers of central nervous system damage: cerebrospinal fluid glial fibrillary acidic protein, neurofilament light chain, and soluble triggering receptor expressed on myeloid cells 2 (*p* < 0.001); plasma phosphorylated tau (*p* = 0.024); and amyloid beta 42/40 ratio (*p* < 0.001). Class 2 experienced more pain (*p* = 0.006) and received more morphine equivalents (*p* = 0.018).

**DISCUSSION:**

Delirium and neighboring phenotypes should be investigated thoroughly in the newly dawning era of precision medicine, to establish novel treatments.

**Highlights:**

- Latent class analysis identified two subphenotypes of patients.
- Both groups contained patients with delirium or its individual symptoms.
- Groups differed by age, education, depression, independent living, and pain levels.
- Groups differed by pre‐operative and post‐operative cognition.
- Groups differed by biomarker levels of neurodegeneration and neuronal injury.

## Graphical abstract

In post‐operative patients undergoing delirium assessment, two latent classes were identified, both containing people experiencing delirium symptoms, but with higher proportion in Class 1. Class 1 were also older, less educated and had more depression. They performed worse in all preoperative cognitive assessments and had more markers of CNS damage. Class 2 experienced more pain and received more morphine equivalents.

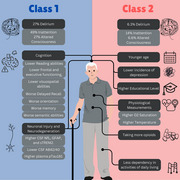

## 1 BACKGROUND

Delirium is a common, transient clinical syndrome characterized by disturbance in attention and cognitive change. The syndrome has heterogenous etiologies and clinical presentations and is often associated with adverse outcomes including acquired dementia, institutionalization, and death.^1^ Currently, delirium is most commonly considered present or absent, based on accepted diagnostic criteria.^2^ This binary classification does not consider the complete patient profile,^3^ lacking full description of symptoms and without consideration for underlying pathophysiology. The pathophysiological mechanisms underlying the clinical symptoms of delirium remain largely hypothetical, with no recommended pharmacological treatments for the syndrome.

To improve understanding of delirium as a syndrome, its clinical presentation, particularly its individual symptoms, must be better defined.^3^ Patients experiencing symptoms of delirium but falling short of the delirium diagnostic criteria might currently be included in the “no delirium” or “control” groups in research studies, therefore compromising the results of analyses. In cases in which symptoms of delirium do not fulfill complete criteria for delirium diagnosis, the term subsyndromal delirium can be used,^4^, ^5^, ^6^ and considered part of a delirium severity spectrum.^7^

Better characterization of the spectrum of delirium syndrome, from those with only individual symptoms, to those with severe delirium, should involve coupling clinical presentation (including individual symptoms and their fluctuation) with underlying pathological mechanisms and exploring associations between them. Mapping delirium symptoms with underlying mechanisms could result in the identification of delirium subphenotypes or endotypes,^8^ expanding understanding of the condition itself, and guiding the development of effective prevention and novel pharmacological treatment strategies.^9^, ^10^, ^11^, ^12^ Such treatments may be informed by the individual patients’ clinical symptoms and biomarkers. Furthermore, it is likely that better understanding of the syndrome will improve delirium diagnosis and prevention by increasing screening, screening accuracy, and risk prediction. Due to the spectrum of disturbance from no delirium, to individual delirium symptoms, to full delirium, it is important to consider those without any symptoms of delirium in analyses.

As research interest in precision medicine increases, particularly in psychiatry,^13^, ^14^ data‐driven probabilistic approaches are empirical to understanding mechanisms of pathophysiology, agnostic to the disease diagnosis.^15^, ^16^ To date, one study has been published using data‐driven subtyping analysis to investigate subtypes of delirium. Potter et al. analyzed a critical care cohort to identify four delirium subtypes which differed in short‐term outcomes, based on indicators of vital signs, laboratory results, and ventilation factors.^17^ Notably, this analysis did not incorporate individual delirium symptoms or biomarkers. Potter et al. used latent class analysis (LCA), a form of probabilistic, finite mixture modeling that allows identification of previously unobserved or unmeasured groups within a population. Previous work using LCA has also been conducted in critical care settings, investigating conditions such as acute respiratory distress syndrome (ARDS),^18^, ^19^ sepsis,^20^ and acute kidney injury.^21^ LCA modeling follows the assumption that the distribution of the indicators in the model is a result of a finite and unobserved mixture of underlying distributions.^22^ For these reasons, LCA is therefore a more statistically robust method of clustering data, compared to more traditional methods such as hierarchal and k‐means clustering,^22^, ^23^, ^24^ which use arbitrary distance measures, introducing inherent subjectiveness and lacking hypothesis.^23^ It has also been shown to have a misclassification approximately four times lower than k‐means[Table alz70516-tbl-0001] clustering.^25^

This paper aims to establish how LCA, carried out using cognitive and biomarker parameters, stratifies an elective orthopedic population, without inclusion of delirium status in the model. By identifying previously unobserved subphenotypes in this population, it is hoped that understanding of delirium syndrome, its neighboring phenotype subsyndromal delirium, and its individual symptoms, will be increased, by exploring associations with biological markers and outcomes. The findings may then inform future investigations on potential underlying mechanisms of delirium, driving development and discovery of novel, targeted treatments for the syndrome.

## 2 METHODS

### 2.1 Study population

This is a secondary analysis of the Postoperative Delirium Belfast (PoDB) observational cohort study, which recruited *n* = 315 participants, aged ≥ 65 years, without a diagnosis of dementia, admitted to hospital for elective primary hip and knee replacement under spinal anesthetic between March 2012 and October 2014.^26^, ^27^ Eligible participants were recruited, and completed baseline assessments, on admission to hospital, either the afternoon before or the morning of surgery. The study was performed in accordance with local ethical committee procedures and all participants gave informed written consent (REC reference: 10/NIR01/5; protocol number: 09069PP‐OPMS). Baseline demographic, cognitive, and perioperative details were collected as described elsewhere.^26^, ^27^ Participants were assessed for delirium and its individual symptoms once daily for the first three post‐operative days using the Confusion Assessment Method (CAM),^28^ supported by the Mini‐Mental State Examination (MMSE),^29^ and nursing staff enquiries. Details of the tests administered are described in Table 1. Post‐discharge nursing and medical notes were interrogated where possible. Samples of cerebrospinal fluid (CSF) and lithium heparin blood plasma were collected from each participant immediately pre‐operatively, as previously described.^26^, ^27^

**TABLE 1 alz70516-tbl-0001:** Description of cognitive and delirium assessments used in this study and how they are scored.

Test	Domains	Scoring
MMSE	Orientation Registration Attention and calculation (WORLD backwards) 3 object recall Language and praxis (naming, repetition, 3‐step command, reading, writing, and copying)	10 points 3 points 5 points 3 points 9 points Total = 30 points
Months of the year backwards	Attention Concentration Working memory	Number of months recited backward correctly before an uncorrected error.
mRASS	Agitation and Sedation Level of alertness	Scored from –5 to +4 based on level of patient sedation, drowsiness, alertness, or agitation.
CAM	Delirium (acute onset, fluctuating course, inattention, disorganized thinking, altered level of consciousness)	Binary score based on presence or absence of four features.

Abbreviations: CAM, Confusion Assessment Method^28^; MMSE, Mini‐Mental State Examination^29^; mRASS, Modified Richmond Agitation and Sedation Scale^34^.

**RESEARCH IN CONTEXT**

1. **Systematic review**: The authors reviewed the literature using traditional sources. Research efforts in understanding the delirium phenotype and its relationship with poor outcomes are increasing but the biology underlying the clinical symptoms of the syndrome remain poorly understood. These works are appropriately cited.
2. **Interpretation**: We identified two subphenotypes of post‐operative patients undergoing delirium assessment. Both groups contained participants with delirium or its individual symptoms. The groups differed significantly by age, educational level, cognition, activities of daily living, and pain levels. They also differed by cerebrospinal fluid and blood plasma biomarkers of neurodegeneration and neuronal injury.
3. **Future directions**: These findings will aid the development of clinical studies for identification of pharmacological treatments of delirium, of which there are currently none. This study supports efforts to move toward precision in the prevention, management, and treatment to increase understanding of the etiologies and underlying mechanisms of delirium.

Biomarker analyses were completed by a trained researcher at University College London. Interleukin (IL)‐1β, IL‐6, IL‐8 and tumor necrosis factor alpha (TNF‐α) concentrations in blood plasma were measured neat by a multiplexed sandwich assay using the Meso Scale Discovery (MSD) V‐PLEX Proinflammatory Panel 1 Human kit and a SECTOR S 600 MM instrument from MSD.^30^ CSF soluble triggering receptor expressed on myeloid cells 2 (sTREM2) concentration was measured with a 4x dilution, using an immunoassay protocol previously described by Banerjee et al.^31^ A Quanterix Neurology 4‐plex E Single molecule array (Simoa) assay was used to measure CSF amyloid beta (Aβ)40, Aβ42, neurofilament light chain (NfL), and glial fibrillary acidic protein (GFAP) concentrations, following the manufacturer's instructions (Quanterix).^32^ Plasma phosphorylated tau (p‐tau)181 concentration was measured using the Quanterix phosphorylated‐tau181 (p‐tau181) advantage kit.^32^ CSF platelet‐derived growth factor receptor beta (PDGFRβ) concentration was measured with a 2x dilution, using Invitrogen Human PDGFR beta ELISA kit, following the manufacturer's instructions.^33^ To reduce potential risks of bias, the analysts conducting the assays at University College London were blinded to patient status from each sample. Samples analyzed using MSD assays, and the Invitrogen assay were measured in duplicate, and those analyzed using Quanterix kits were measured individually, using pooled plasma as internal controls. The pooled inter‐plate percentage coefficients of variance are detailed in Appendix A in supporting information.

### 2.2 Model development

Class‐defining indicators were chosen by selecting variables deemed clinically or biologically relevant to the presentation of delirium syndrome. Individual symptoms of delirium were considered, alongside cognitive measures, co‐morbidities, and biomarkers of inflammation and neurodegeneration. Indicators that were composite of other included indicators in the model were excluded, as were highly correlated variables, unless the rationale for inclusion was clearly stated.

The included indicators were a mix of baseline and post‐operative measures, to incorporate pre‐operative health and cognitive status, as well as peri‐operative change in biological parameters. Full details of included indicators and their abbreviations can be found in Appendix B in supporting information. Notably, we reported individual symptoms of inattention and altered consciousness. Those with inattention were identified as such if they had any new errors peri‐operatively from baseline to day 1 in the “Months of the Year Backwards” (MOTYB) or “World backwards” test, or subjective inattention as tested by the CAM. Those with altered level of consciousness were identified from having a Modified Richmond Agitation and Sedation Scale (mRASS)^34^ score other than 0 on post‐operative day 1, or subjective altered level of consciousness as measured by CAM.

All analyses were completed using R Studio Version 2023.03.1+446 with MPlus version 1.1.8. The full code used to conduct this analysis can be found in Material S1 in supporting information.

### 2.3 Normalization, correlation, and syntax

The distribution of all continuous variables was inspected, and those that were skewed were selected for normalization. The best technique for normalization of the selected values was assessed, and the indicators requiring normalization were normalized using Box–Cox normalization to minimize extreme scales and increase the likelihood that classes would be informative. Continuous variables containing negative numbers were mutated to ensure all values were positive. Pre‐normalization and post‐normalization density plots were generated. Post‐normalization, all continuous variables were centered, scaled, and assessed for correlation. A correlation matrix was assembled using Spearman correlation coefficient, handling missing data using pairwise complete observations. Correlation plots were generated and can be viewed in the Appendices in supporting information. Highly correlated indicators were either included or excluded based on their statistical significance or derivation from one another. After assessment of statistically significant correlations, the Stroop Test (STROOP) and the minimum Diastolic Blood Pressure (MinDBP) were removed from the analysis. National Adult Reading Test (NART) was retained despite its collinearity with Letter Fluency (LETTER), as they are different measures. NART was a baseline measure of premorbid ability, and LETTER is a verbal fluency test. Both LETTER and Category Fluency (CATEG) were retained in the model because they are tests of different functions and are mediated by different areas of the brain.^35^, ^36^ IL‐6, IL‐8, GFAP, and NfL were all retained due to their potential plausibility in allocating mechanistic subphenotypes. The continuous indicators retained in the model were assessed and those requiring normalization were normalized using Box–Cox transformation. After transformation and assessment for correlation, the full dataset was reconstructed.

The dataset was coded in preparation for MPlus (removal of headings and recoding of missing variables), and an MPlus syntax template prepared in a.txt file. An example of the contents of this syntax file is detailed in Material S2 in supporting information. Five Mplus input files were generated from the template file, detailing the code for an LCA defining one to five classes.

### 2.4 Statistical analysis

The LCA was run and models of one to five latent classes were fitted for the cohort. All participants from the PoDB cohort were included. Multiple random starts (*N* = 200) were used to ensure model stability and convergence, of which the best 50 were used for final optimization. The optimal model was selected based on prospectively defined criteria.^22^ These criteria incorporated the Bayesian information criterion (BIC), the Vuong–Lo–Mendell–Rubin (VLMR) likelihood ratio test, and the smallest class size.^22^ The BIC is a measure of how well a model fits, balancing its complexity against sample size, in which a decreasing value indicates better fit.^22^ The VLMR likelihood ratio test measures if *k* number of classes is better than *k* – 1 number of classes. The *p* value presented as a result of the VLMR should also be considered alongside biological and clinical plausibility, when determining the optimal model.^22^ Degree of missingness was small and any missing data were classed as “missing at random.” Estimation of the latent class models was based on full‐information maximum likelihood methods, allowing data from all patients to be used, including those with small volumes of missing data.

Once the optimal model was determined, descriptive statistics were compared among classes. For continuous variables, the Kruskal–Wallis rank sum test was used, and the Fisher test was used for categorical variables. Mean *Z* scores of each continuous variable were calculated and plotted on a line graph to visualize the differences in variables between the subphenotypes. Differences in categorical variables were visualized using bar charts.

### 2.5 Post hoc analysis

Due to the known association of age with risk for delirium, and many of its contributing factors, a post hoc analysis was conducted. This analysis aimed to check if the LCA had split the cohort into the oldest and youngest groups of the population. The PoDB population was split into its older and younger subsections, aligning with the proportions by which the population had been split into latent classes. The proportion of Class 1 members in the older and younger subgroups was determined. Descriptive statistics were generated using the Kruskal–Wallis rank sum test for continuous variables, and the Fisher test for categorical variables. These results were compared to those from the main LCA.

## 3 RESULTS

Details of the analyses conducted to assess correlation and normality of LCA indicators can be found in Appendix C: Table C1, Figures C1 and C2 in supporting information.

Table 2 displays the fit statistics of each model containing two to five classes, for the PoDB cohort. The lowest BIC score was in the three‐class LCA model; however, the VLMR *p* value only showed statistical significance in the two‐class model. In both the two‐class and three‐class models, none of the classes represented < 5% of the population. The entropy levels were high among all classes, indicating high accuracy of patient assignment to the classes. Based on the BIC and VLMR results, the two‐class model was therefore chosen as the most optimal. The two‐class model demonstrated high classification quality, with an entropy of 0.845. The average posterior probabilities for most likely class membership were 0.956 for Class 1 and 0.958 for Class 2, indicating strong separation between the subtypes.

**TABLE 2:** BIC, entropy, class numbers and T11 VLMR *p* value results from latent class analysis investigating five different models.

Number of classes	BIC	Entropy	1	2	3	4	5	T11 VLMR *p* value
2	73809.9	0.836	110	205	*NA*	*NA*	*NA*	0.013
3	73465.5	0.912	77	179	59	*NA*	*NA*	0.60
4	73509.6	0.923	7	83	172	53	*NA*	0.13
5	73619.7	0.931	2	108	135	59	11	0.76

Abbreviations: BIC, Bayesian information criteron; VLMR, Vuong–Lo–Mendell–Rubin likelihood ratio test.

### 3.1 Clinical and biological characteristics of each phenotype

The descriptive statistics and their differences between the two classes are displayed in Table 3. Twenty‐eight percent of Class 1 were delirious, as measured by CAM alongside symptom reports. Class 1 were defined by older age (*p* < 0.001) and less education (*p* < 0.001). Class 1 was characterized by higher Geriatric Depression Scale (GDS) scores (*p* < 0.001), more impairment in Basic Activities of Daily Living (BADL; *p* < 0.001), higher visual vertical analogue score of pain at rest (VVAS; *p* = 0.006), and higher anticholinergic burden (*p* = 0.046). Class 1 had lower scores in their pre‐operative cognitive tests, including the NART (*p* < 0.001), letter fluency (*p* < 0.001), category fluency (*p* < 0.001), color trails 2 (*p* < 0.001), New York Paragraph Recall (NYPR) delayed recall (*p* < 0.001), intersecting pentagons (*p* < 0.001), and orientation tests (*p* < 0.001). Post‐operatively, Class 1 did worse in Three Object Recall (*p* < 0.001), and Three Step Command (*p* < 0.001). Peri‐operatively, some members of Class 1 lost points in their orientation scores, whereas some members gained orientation (*p* < 0.001).

**TABLE 3:** Descriptive statistics for the two‐class Postoperative Delirium Belfast latent class analysis model including indicators from the model and outcome measures.

Characteristic	Class 1, *N* = 110^d^	Class 2, *N* = 205^d^	*p* ^e^	*q* ^f^
**Baseline demographics and clinical characteristics**				
**Age (years)**	78.0 (74.0, 81.8)	71.0 (68.0, 76.0)	**<0.001**	**<0.001**
**Sex**			>0.9	>0.9
Male	47 (43%)	89 (43%)		
Female	63 (57%)	116 (57%)		
**Surgery type**			0.4	>0.9
Hip	51 (46%)	106 (52%)		
Knee	59 (54%)	99 (48%)		
**Years of education**			**<0.001**	**0.02**
6	1 (0.9%)	0 (0%)		
9	1 (0.9%)	2 (1.0%)		
10	76 (69%)	44 (22%)		
11	16 (15%)	51 (25%)		
12	8 (7.3%)	33 (16%)		
13	5 (4.5%)	20 (9.9%)		
14	1 (0.9%)	15 (7.4%)		
15	0 (0%)	4 (2.0%)		
17	2 (1.8%)	33 (16%)		
**Diabetes**			0.2	>0.9
No	84 (83%)	170 (89%)		
Yes	17 (17%)	22 (11%)		
**Hypertension**			0.3	>0.9
No	35 (35%)	82 (41%)		
Yes	66 (65%)	116 (59%)		
GDS^c^	3.00 (1.00, 5.00)	2.00 (1.00, 3.00)	**<0.001**	**0.007**
BADL^c^	3.00 (1.00, 3.00)	2.00 (1.00, 3.00)	**<0.001**	**0.034**
VVAS at rest^c^	17 (5, 42)	31 (10, 57)	**0.006**	0.2
NART score^c^	21 (14, 27)	33 (25, 39)	**<0.001**	**<0.001**
**ACB**			**0.046**	>0.9
0	38 (37%)	71 (37%)		
1	29 (28%)	78 (41%)		
2	18 (18%)	19 (10%)		
3	4 (3.9%)	10 (5.3%)		
4	6 (5.9%)	5 (2.6%)		
5	2 (2.0%)	6 (3.2%)		
6	2 (2.0%)	0 (0%)		
7	2 (2.0%)	1 (0.5%)		
8	1 (1.0%)	0 (0%)		
**ASA status**			0.094	>0.9
1	2 (1.9%)	13 (6.3%)		
2	84 (79%)	166 (81%)		
3	20 (19%)	26 (13%)		
**Cognition**				
Letter fluency^c^	7.8 (6.0, 10.7)	13.3 (10.3, 16.0)	**<0.001**	**<0.001**
Category fluency^c^	13.5 (11.5, 16.5)	18.0 (16.0, 21.0)	**<0.001**	**<0.001**
Color trails 2 time^c^	197 (161, 252)	118 (99, 146)	**<0.001**	**<0.001**
NYPR delayed recall^c^	4.00 (2.00, 5.00)	6.00 (4.00, 7.00)	**<0.001**	**<0.001**
**Intersecting pentagons score** ^c^			**<0.001**	**<0.001**
0	46 (42%)	24 (12%)		
1	64 (58%)	180 (88%)		
**Orientation score** ^c^			**<0.001**	**0.041**
3	1 (0.9%)	0 (0%)		
5	1 (0.9%)	0 (0%)		
8	3 (2.7%)	0 (0%)		
9	26 (24%)	27 (13%)		
10	79 (72%)	178 (87%)		
**Orientation change** ^a^			**<0.001**	**0.02**
−3	1 (0.9%)	0 (0%)		
−2	1 (0.9%)	0 (0%)		
−1	13 (12%)	17 (8.6%)		
0	59 (54%)	165 (83%)		
1	27 (25%)	15 (7.6%)		
2	3 (2.7%)	1 (0.5%)		
3	5 (4.5%)	0 (0%)		
4	1 (0.9%)	0 (0%)		
**Three object recall score** ^b^			**<0.001**	**0.02**
0	13 (12%)	2 (1.0%)		
1	28 (25%)	13 (6.6%)		
2	39 (35%)	78 (39%)		
3	30 (27%)	105 (53%)		
**Three step command score** ^b^			**0.003**	0.14
1	8 (7.3%)	3 (1.5%)		
2	24 (22%)	27 (14%)		
3	77 (71%)	165 (85%)		
**Physiological measures**				
Min SpO2^b^	95.00 (95.00, 96.00)	96.00 (95.00, 96.25)	**0.009**	0.3
Max Temp^b^	37.40 (37.10, 37.80)	37.70 (37.20, 37.90)	**0.014**	0.6
Min SBP^b^	97 (90, 105)	99 (91, 106)	0.4	>0.9
Max HR^b^	86 (78, 92)	88 (82, 97)	0.094	>0.9
Total Meq^b^	18 (12, 31)	27 (15, 34)	**0.018**	0.7
**Biomarkers**				
IL‐1b^a^	0 (−68, 8)	0 (−70, 0)	0.8	>0.9
IL‐6^a^	3,157 (1,051, 6,659)	3,471 (1,514, 11,502)	0.073	>0.9
IL‐8^a^	771 (374, 1,452)	972 (464, 2,028)	0.12	>0.9
TNF‐α^a^	−9 (−48, 45)	−2 (−38, 49)	0.3	>0.9
Aβ42/40 ratio	0.050 (0.030, 0.070)	0.070 (0.050, 0.090)	**<0.001**	**<0.001**
CSF GFAP^c^	9,778 (7,502, 13,242)	6,294 (4,594, 8,749)	**<0.001**	**<0.001**
CSF NfL^c^	1,613 (1,219, 2,065)	1,022 (774, 1,401)	**<0.001**	**<0.001**
CSF sTREM2^c^	3,620 (3,026, 4,272)	3,013 (2,512, 3,771)	**<0.001**	**0.013**
CSF PDGFRβ^c^	432 (353, 511)	427 (364, 508)	>0.9	>0.9
Plasma p‐tau181^c^	4.00 (2.57, 5.57)	3.24 (2.16, 5.07)	**0.024**	>0.9
Serum CSF albumin ratio^c^	5.29 (4.04, 7.74)	5.18 (4.09, 7.00)	0.4	>0.9
**Delirium and individual symptoms**				
**Delirium by CAM**			**0.012**	0.5
No	98 (89%)	198 (97%)		
Yes	12 (11%)	7 (3.4%)		
**Delirium by CAM and reports**			**<0.001**	**<0.001**
No	79 (72%)	192 (94%)		
Yes	31 (28%)	13 (6.3%)		
**Inattention** ^b^			**<0.001**	**<0.001**
No	56 (51%)	170 (86%)		
Yes	54 (49%)	28 (14%)		
**Altered level of consciousness** ^b^		**<0.001**	**<0.001**	
No	80 (73%)	185 (93%)		
Yes	30 (27%)	13 (6.6%)		

Abbreviations: Aβ, amyloid beta; ACB, anticholinergic burden; ASA, American Society of Anesthesiologists Physical Status Classification System; BADL, Basic Activities of Daily Living; CAM, Confusion Assessment Method; CSF, cerebrospinal fluid; GDS, Geriatric Depression Scale; GFAP, glial fibrillary acidic protein; HR, heart rate; IL, interleukin; IQR, interquartile range; Max, maximum; Meq, morphine equivalents; in, minimum; *N*, number; NART, National Adult Reading Test; NfL, neurofilament light chain; NYPR, New York Paragraph Recall; PDGFRβ, platelet‐derived growth factor receptor beta; p‐tau, phosphorylated tau; SBP, systolic blood pressure; sTREM2, soluble triggering receptor expressed on myeloid cells 2; Temp, temperature; TNF‐α, tumor necrosis factor alpha; VVAS, Visual Vertical Analogue Score of Pain at Rest.

Bold values shows *p* < 0.05.

^a^ Peri‐operative change.

^b^ Post‐operative.

^c^ Pre‐operative.

^d^ Median (IQR); *n* (%).

^e^ Wilcoxon rank sum test; Fisher exact test; Fisher exact test for count data with simulated *p* value (based on 2000 replicates).

^f^ Bonferroni correction for multiple testing.

**FIGURE 1 alz70516-fig-0001:**
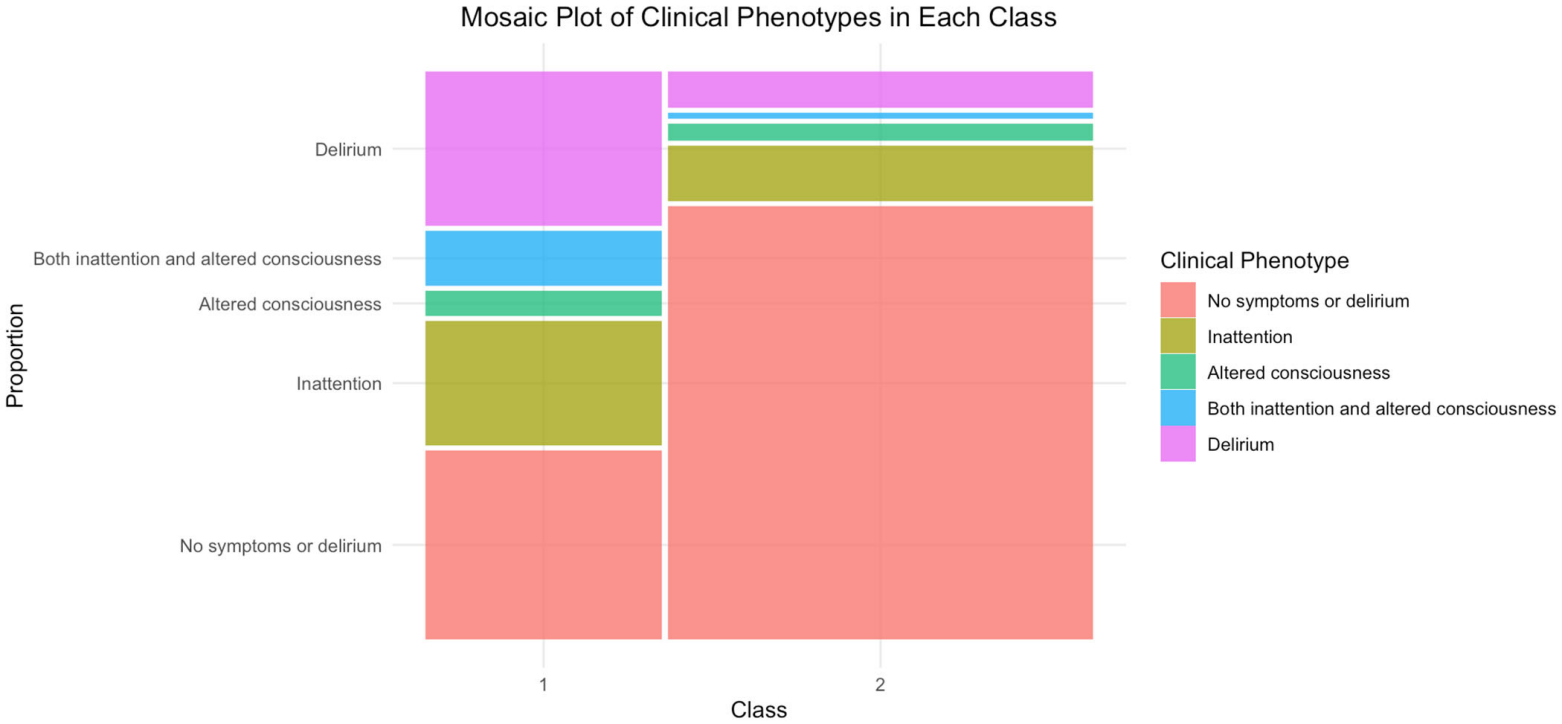
A mosaic plot displaying the distribution of occurrences of individual delirium symptoms (inattention and altered consciousness) and full delirium across the two latent classes.

Class 1 is set apart by higher incidence of individual delirium symptoms, whereby 49% of Class 1 experienced post‐operative inattention (*p* < 0.001), and 27% experienced altered level of consciousness (*p* < 0.001). The distribution of these clinical phenotypes across the two classes can be observed in Figure 1. Figure 1 clearly demonstrates that a much larger proportion of Class 2 had no symptoms at all, compared to Class 1.

Physiologically, Class 1 is distinguishable by their lower peripheral oxygen saturation (SpO_2_; *p* = 0.009), despite its remaining within normal levels. The overall physiological results of Class 1 are also differentiated by lower median score of maximum temperature and lower number of total morphine equivalents (TMeqs; *p* = 0.014). The median pre‐operative biomarkers of Class 1 were identifiable by high CSF GFAP (*p* < 0.001), NfL (*p* < 0.001), and sTREM2 (*p* < 0.001), as well as high pre‐operative plasma p‐tau181 (*p* = 0.024), Class 1 was further distinguished by a lower CSF Aβ42/40 ratio (*p* < 0.001).

In Class 2, 6.3% of individuals were delirious, 14% experienced post‐operative inattention, and 6.6% had an altered level of consciousness. Similarly to Class 1, Class 2 lost points in orientation peri‐operatively, but Class 2 had a greater proportion of individuals with consistent orientation scores. Class 2 were overall characterized by performing better cognitively and physiologically, compared to Class 1.

**FIGURE 2 alz70516-fig-0002:**
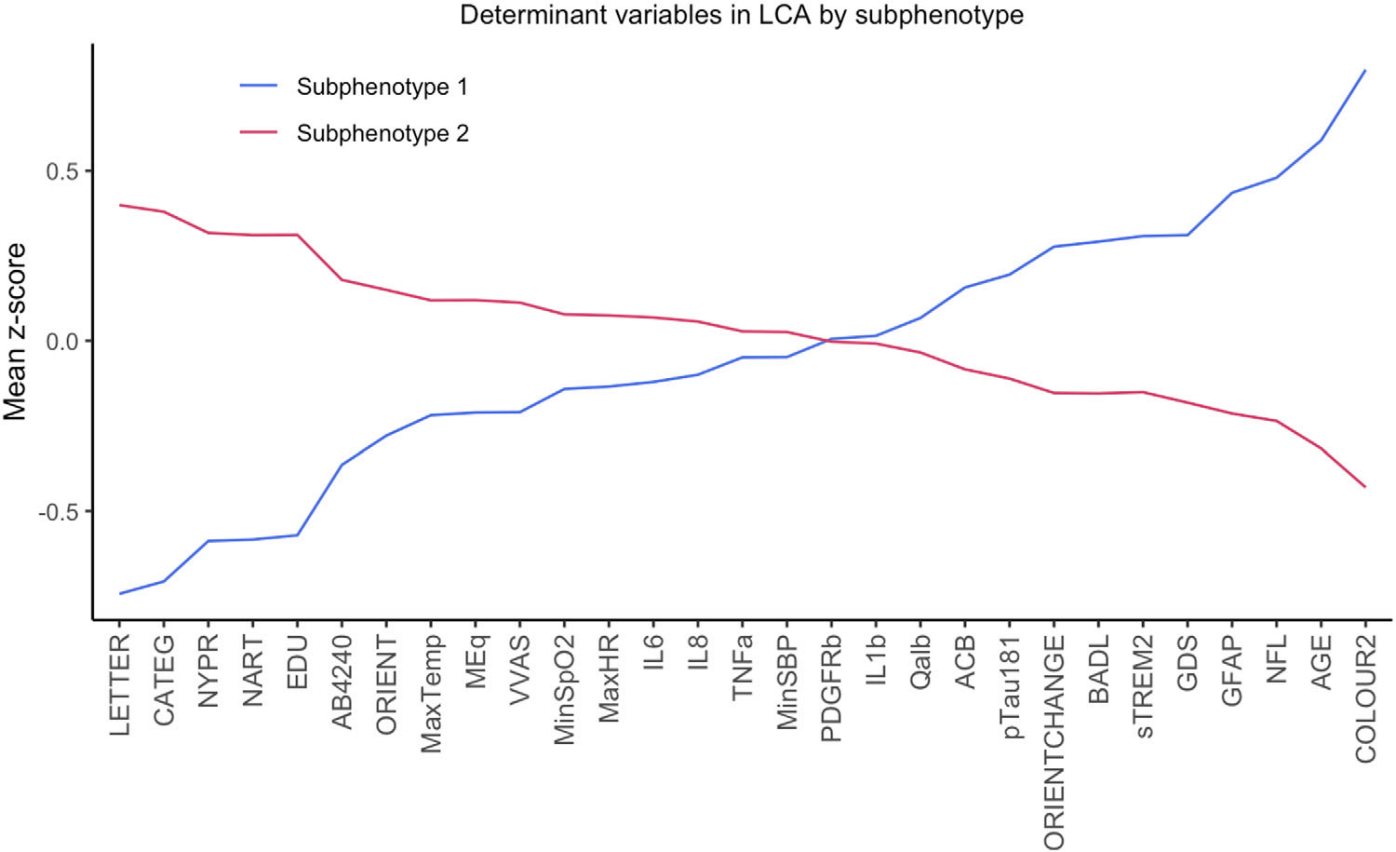
A line graph of the two identified classes or subphenotypes, separated by the mean *z* score of each continuous variable. LCA, latent class analysis

**FIGURE 3 alz70516-fig-0003:**
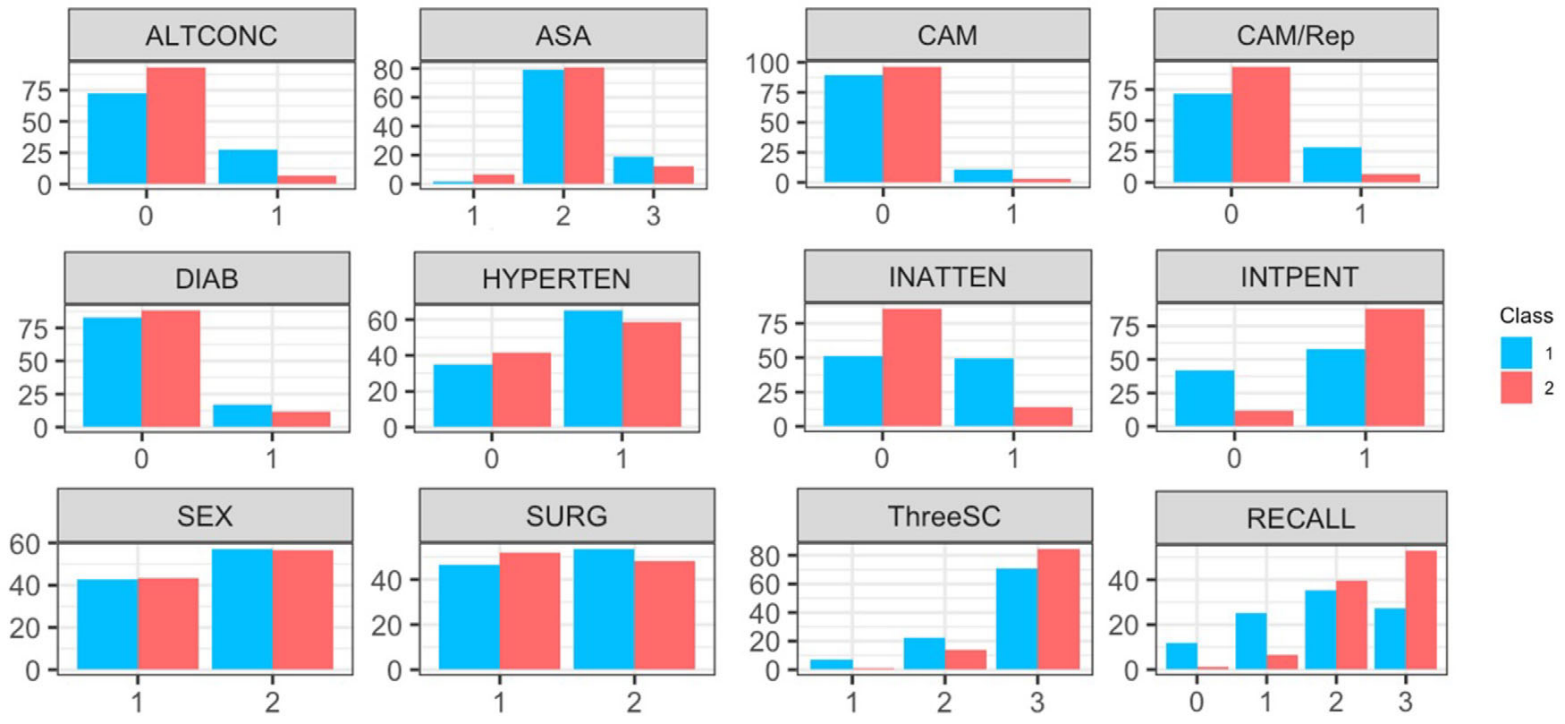
Bar charts showing the difference in the proportion of participants falling into each category between the two classes. ALTCON, Altered Level of Consciousness; ASA, American Society of Anesthesiologists Physical Status Classification System; CAM, Confusion Assessment Method; CAM/Rep, Confusion Assessment Method and reports; DIAB, diabetes; HYPERTEN, hypertension; INATTEN, inattention; INTPENT, Intersecting Pentagons; SURG, Type of Surgery; ThreeSC, Three Step Command.

The separation in classes is clearly displayed by Figure 2—a line graph showing the mean *z* score for each of the continuous variables included in the model.^18^ The difference in categorical variables between the two classes is visually demonstrated by the bar charts in Figure 3.

### 3.2 Post hoc analysis

Due[Fig alz70516-fig-0001], [Fig alz70516-fig-0002], [Fig alz70516-fig-0003] to the large difference in median age between the two PoDB classes, it is possible that although age did not show significant correlations with the other indicators, it could be a contributing factor to the other significant findings within the model. Post hoc analyses were carried out to investigate if these differences were also significant when the oldest group of the population, and Class 1, were compared. As Class 1 had a population of 110, the PoDB cohort was broken down into the oldest 110 and the younger 205 people. Results of this analysis are shown in Table 4.

**TABLE 4 alz70516-tbl-0004:** Results of the PoDB post hoc analyses comparing the older 110 people in the cohort, and Class 1.

Characteristic	Older group, *N* = 110^d^	Class 1, *N* = 110^e^	*p* ^e^	*q* ^f^
**Baseline demographics and clinical characteristics**				
**Age (years)**	80.0 (78.0, 83.0)	78.0 (74.0, 81.8)	**<0.001**	**<0.001**
**Sex**			0.7	>0.9
Male	43 (39%)	51 (46%)		
Female	67 (61%)	63 (57%)		
**Surgery type**			>0.9	>0.9
Hip	52 (47%)	106 (52%)		
Knee	58 (53%)	59 (54%)		
**Years of education**			**0.033**	<0.9
6	0 (0%)	1 (0.9%)		
9	2 (1.8%)	1 (0.9%)		
10	60 (55%)	76 (69%)		
11	12 (11%)	16 (15%)		
12	11 (10%)	8 (7.3%)		
13	7 (6.4%)	5 (4.5%)		
14	3 (2.8%)	1 (0.9%)		
15	1 (0.9%)	0 (0%)		
17	13 (12%)	2 (1.8%)		
**Diabetes**			0.7	>0.9
No	96 (97%)	93 (85%)		
Yes	14 (13%)	17 (15%)		
**Hypertension**			0.7	>0.9
No	75 (35%)	44 (40%)		
Yes	71 (65%)	66 (60%)		
GDS^c^	2.00 (1.00, 4.00)	3.00 (1.00, 5.00)	0.082	>0.9
BADL^c^	3.00 (2.00, 3.00)	3.00 (1.00, 3.00)	0.8	>0.9
VVAS at rest^c^	18 (4, 52)	17 (5, 42)	0.7	>0.9
NART score^c^	28 (21, 37)	21 (14, 27)	**<0.001**	**<0.001**
**ACB**			>0.9	>0.9
0	40 (40%)	38 (37%)		
1	32 (32%)	29 (28%)		
2	15 (15%)	18 (18%)		
3	4 (4%)	4 (4%)		
4	5 (5%)	6 (6%)		
5	3 (3.0%)	2 (2%)		
6	1 (1.0%)	2 (2%)		
7	1 (1.0%)	2 (2%)		
8	0 (0%)	1 (1%)		
**ASA status**			>0.9	>0.9
1	3 (2.8%)	2 (1.9%)		
2	84 (78%)	84 (79%)		
3	21 (19%)	20 (19%)		
**Cognition**				
Letter fluency^c^	10.7 (7.4, 13.2)	7.8 (6.0, 10.7)	**<0.001**	0.003
Category fluency^c^	14.8 (12.5, 17.5)	13.5 (11.5, 16.5)	**0.042**	>0.9
Color trails 2 time^c^	179 (135, 235)	197 (161, 252)	**0.024**	>0.9
NYPR delayed recall^c^	4.00 (2.00, 6.00)	4.00 (2.00, 5.00)	**0.012**	0.5
**Intersecting pentagons score** ^c^			**0.033**	>0.9
0	79 (72%)	64 (58%)		
1	31 (28%)	46 (42%)		
**Orientation score** ^c^			0.9	>0.9
3	1 (0.9%)	1 (0.9%)		
5	1 (0.9%)	1 (0.9%)		
8	1 (0.9%)	3 (2.7%)		
9	24 (22%)	26 (24%)		
10	83 (75%)	79 (72%)		
**Orientation change** ^a^			0.9	>0.9
−3	1 (0.9%)	1 (0.9%)		
−2	0 (0%)	1 (0.9%)		
−1	11 (10%)	13 (12%)		
0	69 (63%)	59 (54%)		
1	23 (21%)	27 (25%)		
2	3 (2.7%)	3 (2.7%)		
3	3 (2.7%)	5 (4.5%)		
4	0 (0%)	1 (0.9%)		
**Three object recall score** ^b^			0.2	>0.9
0	10 (9.1%)	13 (12%)		
1	18 (16%)	28 (25%)		
2	40 (36%)	39 (35%)		
3	42 (38%)	30 (27%)		
**Three step command score** ^b^			0.6	>0.9
1	5 (4.6%)	8 (7.3%)		
2	27 (25%)	24 (22%)		
3	77 (71%)	77 (71%)		
**Physiological measures**				
Min SpO2^b^	96.00 (95.00, 96.00)	95.00 (95.00, 96.00)	>0.9	>0.9
Max Temp^b^	37.40 (37.10, 37.80)	37.40 (37.10, 37.80)	0.8	>0.9
Min SBP^b^	96 (90, 104)	97 (90, 105)	0.7	>0.9
Max HR^b^	87 (79, 97)	86 (78, 92)	0.5	>0.9
Total Meq^b^	17 (12, 30)	18 (12, 31)	0.7	>0.9
**Biomarkers**				
IL1b^a^	0 (−88, 46)	0 (−68, 8)	0.6	>0.9
IL6^a^	2,597 (989, 7,314)	3,157 (1,051, 6,659)	>0.9	>0.9
IL8^a^	725 (267, 1,354)	771 (374, 1,452)	0.5	>0.9
TNF‐α^a^	−15 (−55, 43)	−9 (−48, 45)	0.6	>0.9
Aβ42/40 ratio	0.060 (0.040, 0.080)	0.050 (0.030, 0.070)	0.3	>0.9
CSF GFAP^c^	9,214 (6,533, 12,166)	9,778 (7,502, 13,242)	0.3	>0.9
CSF NfL^c^	1,427 (1,016, 2,012)	1,613 (1,219, 2,065)	0.3	>0.9
CSF sTREM2^c^	3,643 (3,036, 4,786)	3,620 (3,026, 4,272)	0.4	>0.9
CSF PDGFRβ^c^	439 (371, 514)	432 (353, 511)	0.5	>0.9
Plasma p‐tau181^c^	3.91 (2.45, 5.70)	4.00 (2.57, 5.57)	>0.9	>0.9
Serum CSF albumin ratio^c^	5.17 (4.07, 7.17)	5.29 (4.04, 7.74)	0.5	>0.9
**Delirium and individual symptoms**				
**Delirium by CAM**			0.3	>0.9
No	103 (93.6%)	98 (89%)		
Yes	7 (6.4%)	12 (11%)		
**Delirium by CAM and reports**			0.4	>0.9
No	85 (77%)	79 (72%)		
Yes	25 (23%)	31 (28%)		
**Inattention** ^b^			**0.013**	0.6
No	75 (68%)	56 (51%)		
Yes	35 (32%)	54 (49%)		
**Altered level of consciousness** ^b^			0.1	>0.9
No	91 (83%)	80 (73%)		
Yes	19 (17%)	30 (27%)		

Abbreviations: Aβ, amyloid beta; ACB, anticholinergic burden; ASA, American Society of Anesthesiologists Physical Status Classification System; BADL, Basic Activities of Daily Living; CAM, Confusion Assessment Method; CSF, cerebrospinal fluid; GDS, Geriatric Depression Scale; GFAP, glial fibrillary acidic protein; HR, heart rate; IL, interleukin; IQR, interquartile range; Max, maximum; Meq, morphine equivalents; in, minimum; *N*, number; NART, National Adult Reading Test; NfL, neurofilament light chain; NYPR, New York Paragraph Recall; PDGFRβ, platelet‐derived growth factor receptor beta; p‐tau, phosphorylated tau; SBP, systolic blood pressure; sTREM2, soluble triggering receptor expressed on myeloid cells 2; Temp, temperature; TNF‐α, tumor necrosis factor alpha; VVAS, visual vertical analogue score.

Bold values shows *p* < 0.05.

^a^ Peri‐operative change.

^b^ Post‐operative.

^c^ Pre‐operative.

^d^ Median (IQR); *n* (%),

^e^ Wilcoxon rank sum test; Fisher exact test; Fisher exact test for count data with simulated *p* value (based on 2000 replicates),

^f^ Bonferroni correction for multiple testing, *N* = number,

In Class 1, 66 people fell into the older group, so Class 1 members made up 60% of the oldest constituents of the PoDB cohort. This indicated that being older is not the only factor contributing to the poorer results and outcomes of Class 1. Class 1 had a lower median age of 78 compared to 80 (*p* < 0.001) and fewer years of education (*p* = 0.033). Class 1 also had worse performances on NART (*p* < 0.001), letter fluency (*p* < 0.001), category fluency (*p* = 0.042), color trails 2 (*p* = 0.024), and intersecting pentagons (*p* = 0.033). The median NYPR score was the same in the two groups, but the interquartile range was larger in the older group (*p* = 0.012). The symptom of inattention was more common in Class 1 than in the older group (*p* = 0.013).

The remaining indicators included in the LCA were not significantly different between the older group and Class 1. These results show that the poorer cognition scores and the higher occurrence of inattention seen in Class 1 were not only attributable to the higher age of the group. However, based on biomarker levels and outcome data, Class 1 was not significantly different to the older proportion of the PoDB cohort.

## 4 DISCUSSION

LCA was used to identify two population subphenotypes of older elective surgical patients from the cohort, containing 205 and 110 participants, with 28% and 6.3% delirious patients, respectively. In classes 1 and 2, 49% and 14% of participants experienced inattention, and 27% and 6.6% experienced altered level of consciousness, respectively. This is the first analysis of a population being assessed for delirium that used both clinical features and biomarkers to identify subphenotypes.

Class[Table alz70516-tbl-0004] 1 was older, had less time in education, had higher depression scores, dependency in activities of daily living, and higher pain levels. Based on the results from their pre‐operative cognitive tests, they also had lower reading abilities, frontal and executive functioning, visuospatial abilities, memory, orientation, semantic abilities, and worse delayed recall compared to Class 2. Class 1 also did worse in their post‐operative cognitive and delirium tests showing worse memory, more inattention, and more participants with altered levels of consciousness. Physiologically, they had lower oxygen saturation, temperature, and lower numbers of total morphine equivalents. Class 1 participants had higher pre‐operative CSF levels of GFAP, NfL, and sTREM2, indicating higher levels of neuronal injury or neurodegeneration than in Class 2. They also had higher levels plasma p‐tau181 and lower CSF Aβ42/40 ratios, which are biomarkers of amyloid deposition. None of the measures of peri‐operative change in inflammation were statistically different between the two classes. These results clearly show that Class 1 was more vulnerable, based on cognitive, physiological, and biomarker tests.

It is notable that the LCA did not simply separate the population into those with and without delirium, highlighting once again that the syndrome is more complex than a simple binary classification can account for. Indeed, delirium existed in both subtypes, as did its individual symptoms of inattention and altered levels of consciousness. The large number of individuals in Class 2 without any symptoms of delirium may indicate that the latent classes observed in this paper are subtypes of perioperative cognition, rather than delirium. These results highlight the importance of measuring individual symptoms of the syndrome, to increase precision in patient management. Consistent collection of symptom data, alongside biomarker measurement, will provide telling information about the natural history of each individual delirium occurrence.

As this is an elective surgical cohort without pre‐existing dementia or delirium, we know that the insult patients have experienced is surgery. This insult is known, consistent, and controlled, and therefore useful for interpreting the different characteristics of the classes. It is possible that different mechanisms underlie delirium in Classes 1 and 2. For example, those in Class 1 may experience delirium due to an underlying neurodegenerative process, as displayed by their plasma and CSF biomarker levels. On the other hand, the acute insult caused by the surgical process, levels of pre‐operative pain, and post‐operative morphine equivalents, may contribute toward delirium in Class 2. The results of the sensitivity analyses for the PoDB LCA revealed the older constituent of the cohort shared many similarities with those in Class 1, where the median age was 8 years more than Class 2. However, there were also many differences between the older group and Class 1. Age is a known risk factor for delirium,^37^ and will be a largely unavoidable confounder in analyses of this nature. The mechanisms of aging should not be considered separately. Correlation analyses did not indicate that age had statistically significant correlations with other included indicators, and 40% of the oldest constituents were not included in Class 1. For these reasons, the two‐class subphenotype model for PoDB continued to be supported as the optimal model.

The Potter et al. subphenotyping analysis of delirium in the intensive care unit (ICU) found a four‐class model to be an optimal fit.^38^ Of note, Potter et al. analyzed only patients with a positive CAM‐ICU assessment, which differs from the current paper, which includes patients undergoing delirium assessments. Potter et al. also included different candidate variables, focusing on parameters of sedation, ventilation, and medical and laboratory results.^38^ The current paper focused on individual symptoms of delirium, as well as biomarkers of inflammation, neurodegeneration, and neuronal damage, measured from the blood and CSF. The defining features of the classes in both articles are therefore not consistent. These differing approaches to subtyping reflect the differing populations in which the analyses were run—their candidate variables differ depending on available data, ability to screen and interact with patients, and the clinical utility of the measurements. Future work should include comparing and validating the post‐operative cohort in patients with a formal delirium diagnosis or subsyndromal delirium diagnosis to differentiate the populations addressed in each of these papers.

We suggest the findings of this study should be replicated in different and larger cohorts for validation. They should also be validated by development of parsimonious models, using a smaller number of indicators in the LCA. These results will guide future, similar analyses through choice of indicators and methods for carrying out delirium subphenotyping. Future work should also determine the associations of newly identified subtypes with external factors like clinical outcomes, like trajectory and mortality, for validation.^39^

### 4.1 Strengths and limitations

This project is the first to use VCA to investigate subphenotypes of a population being assessed for delirium using both clinical features and biomarkers. The PoDB cohort has well‐described symptom data, and LCA is a powerful data clustering technique that allows inclusion of multiple data types.

These findings also have limitations. This analysis relied on the availability of existing data; therefore, only readily available variables were available for use. Selection of the most appropriate model following LCA may be approached differently by various researchers, despite the existence of criteria such as the BIC and VLMR. The importance of BIC and entropy may also be implemented differently by researchers. BIC is a measure of how well a model fits, balancing model complexity with sample size. It is calculated using the maximum likelihood estimate and penalizes models as the number of indicators increase, so a lower BIC denotes a better fit. Alternatively, entropy is a measure of separation between the latent classes, wherein higher entropy denotes better separation. It is calculated using sample size, number of classes, and posterior probabilities; however, entropy is not primarily used for model selection because an over‐fit model will have a high entropy. For this reason, BIC and VLMR were prioritized during model selection in this paper, and the entropy measure used to support the decisions.

Furthermore, the three‐class model had the lowest BIC, but did not have a VLMR *p* value of < 0.05. For this reason, the two‐class model was selected; however, it is possible that the three‐class model may be more clinically useful in a larger dataset, and the VLMR was statistically significant. This limitation reinforces the need for repetition of this work in larger cohorts.

Steps were taken to exclude those with pre‐existent dementia from the observational study—that is, those with a pre‐existing dementia diagnosis were excluded. However, there remains a small possibility that those with an unidentified and undiagnosed active dementia were included in the analysis and may have impacted class distribution. It seems likely that participants with undiagnosed dementia‐causing diseases are included, as is the case in all studies involving older people. Indeed, this work suggests, in line with existing evidence, that latent dementia‐causing diseases, or neurodegeneration, are a significant risk factor for delirium symptoms. While the authors aimed to select the most appropriate and informative assessments of cognition, delirium, and individual symptoms of delirium, there remains the chance that different assessment tools may have produced different results. This should be considered during future research. Furthermore, this work advances delirium subtyping science due to the inclusion of new variables (biomarkers), interpretation of phenotypes should be done with caution, particularly in relation to whether these new groups reflect “delirium” or “peri‐operative cognition” subtypes.

## 5 CONCLUSIONS

Latent class analyses were completed to identify two population subphenotypes. The two subphenotypes differed in age, education levels, depression, activities of daily living, pain levels, pre‐operative cognition, and post‐operative performance in cognitive assessments. They also varied in CSF biomarker levels of neurodegeneration and neuronal injury, GFAP, NfL, and sTREM2, and the plasma inflammatory marker p‐tau181. Delirium (*p* < 0.001), inattention (*p* < 0.001), and altered consciousness (*p* < 0.001), occurred in both classes, but more so in the subphenotype with poorer performance in most indicators. These results clearly indicate that Class 1 is a phenotype of greater vulnerability. These findings require repetition and validation in other cohorts; however, they show that there are previously unobserved latent classes in these populations based on clinical and biomarker data. Further investigation into population subphenotypes in those at risk for delirium may increase understanding of the underlying mechanisms of delirium, and drive research for identification of pharmacological treatments. Furthermore, development of parsimonious models of delirium subphenotype identification may have applicability for future endeavors in bedside subphenotype assignment using clinical features or biomarkers.

## AUTHOR CONTRIBUTIONS

*Funding acquisition and conceptualization*: Emma L. Cunningham, Daniel F. McAuley, Bernadette McGuinness, and Anthony P. Passmore. *Data collection*: Emma L Cunningham, David Beverland, Henrik Zetterberg, Jonathan M. Schott, Amanda Heslegrave, Elena Veleva, and Rhiannon Laban. *Project conception*: Emily Margaret Louise Bowman, Emma L. Cunningham, and Daniel F McAuley. *Methodology*: Emily Margaret Louise Bowman, Emma L. Cunningham, and Daniel F. McAuley. *Data analysis*: Emily Margaret Louise Bowman. *Drafting of manuscript*: Emily Margaret Louise Bowman and Emma L. Cunningham. *Review*: Daniel F. McAuley, Bernadette McGuinness, Anthony P. Passmore, David Beverland, Henrik Zetterberg, Jonathan M. Schott, Amanda Heslegrave, Elena Veleva, Rhiannon Laban, Aoife M. Sweeney, and Emma L. Cunningham.

## CONFLICT OF INTEREST STATEMENT

E.L.C. has received grant funding from Alzheimer's Research UK. H.Z. is a Wallenberg Scholar and a Distinguished Professor at the Swedish Research Council supported by grants from the Swedish Research Council (#2023‐00356, #2022‐01018 and #2019‐02397), the European Union's Horizon Europe research and innovation programme under grant agreement No 101053962, Swedish State Support for Clinical Research (#ALFGBG‐71320), the National Institute for Health and Care Research University College London Hospitals Biomedical Research Centre, and the UK Dementia Research Institute at UCL (UKDRI‐1003). All other authors declare that they have no competing interests. Author disclosures are available in the supporting information.

## CONSENT STATEMENT

Informed consent was obtained from all human subjects.

## Supporting information



Supporting Information

Supporting Information

Appendix Table A

Appendix Table B
Appendix Section C – Description of correlation analysis.
The Correlation Matrix was calculated using the Spearman method. These correlations are also shown in the dot plot. The post‐normalisation density plots are shown below.

Appendix Table C1

Appendix Figure C1: Correlation circle plot displaying correlations between indicators for inclusion in the LCA model.

Appendix Figure C2: Post normalisation density plots for the PoDB indicators.
