# Supplementary material for: Identifying subphenotypes of patients undergoing post‐operative delirium assessment
Source: Alzheimers Dement. 2025 Jul 16;21(7):e70516. doi: 10.1002/alz.70516 (PMC12265012; doi:10.1002/alz.70516)
Supplement: Supplementary file 1 — Supporting Information [file ALZ-21-e70516-s005.docx]

**Supplementary Material**

1. **R Code**

#Determine and set working directory

setwd("")

#Setup- load data and packages

library(tidyverse)

library(bestNormalize)

library(colorspace)

library(corrplot)

library(correlation)

library(MplusAutomation)

library(plyr)

library(plotrix)

library(glue)

library(english)

library(gtsummary)

library(ggpubr)

library(lavaan)

library(purrr)

library(readr)

library(tibble)

library(tidyr)

library(ggplot2)

library(BiocManager)

library(rhdf5)

library(dplyr)

library(viridis)

source("functions")

# load dataset

data <- read_csv ("dataset", na = ".")

#Preprocessing for LCA (Normalisation and correlations)

# set seed

set.seed(1998)

# select continuous variables to transform.(And mutate variables containing negative numbers so normalisation can be completed)

cont_vars <- data %>%

dplyr::select(-c( PT_ID,

SEX,

SURG,

DIAB,

HYPERTEN,

INTPENT,

ASA,

RECALL,

ThreeSC,

INATTEN,

ALTCONC,

CAM,

`CAM/Rep`,

LiveSit,

DEAD,

Cognition)) %>%

mutate(BADL = BADL +1,

VVAS = VVAS +1,

NYPR = NYPR +1,

ACB = ACB +1,

GDS = GDS + 1,

MEq = MEq +1,

ORIENTCHANGE = ORIENTCHANGE + 10,

IL1b = IL1b + 100,

IL6 = IL6 + 100,

IL8 = IL8 + 100,

TNFa = TNFa + 100)

# prenormalisation density plots

prenorm_dplots <- cont_vars %>%

pivot_longer(cols = everything(), names_to = "key") %>%

ggplot(aes(value)) +

geom_density(fill = "#fc9272", color = "#de2d26", alpha = 0.8) +

facet_wrap(~key, scales = "free") +

ggtitle("Prenormalisation density plots") +

theme_classic()

all_numeric <- sapply(data, is.numeric)

# Check if all variables are numeric

if (all(all_numeric)) {

print("All variables are numeric.")

} else {

non_numeric_vars <- names(data)[!all_numeric]

print(paste("Non-numeric variables:",

paste(non_numeric_vars, collapse = ", ")))

}

# use 'bestnorm_table' function to assess for best normalisation technique. Should use the technique with the lowest P. Table shows that Box-Cox normalisation is generally good.

# Therefore use this in all cases where 'center_scale' (i.e. no normalisation) is not superior.

print(bestnorm_table <-bestnorm_table(cont_vars))

# select variables to normalise and apply Box-Cox transformation.

norm_vars <- cont_vars %>%

select(-c("ACB", "ORIENT", "ORIENTCHANGE", "MaxTemp")) %>%

map_df(~boxcox(.x, standardize = FALSE)$x.t)

# select variables from 'cont_vars' that were not transformed and join with variables post Box-Cox transformation

postnorm_cont_vars <- cbind(cont_vars[, -which(names(cont_vars) %in% colnames

(norm_vars))], norm_vars)

# postnormalisation density plots

postnorm_dplots <- postnorm_cont_vars %>%

pivot_longer(cols = everything(), names_to = "key") %>%

ggplot(aes(value)) +

geom_density(fill = "#fc9272", color = "#de2d26", alpha = 0.8) +

facet_wrap(~key, scales = "free") +

ggtitle("Postnormalisation density plots") +

theme_classic() +

theme(

text = element_text(size = 8), # Adjust the font size

plot.title = element_text(size = 12, hjust = 0.5), # Adjust the title font size

strip.text = element_text(size = 6) # Adjust the facet label font size

)

postnorm_dplots

# centre and scale continuous data and save as 'postnorm_postscale_cont_vars'

postnorm_postscale_cont_vars <- data.frame(apply(postnorm_cont_vars, 2, scale))

# construct correlation matrix for indicator variables, uses spearman correlation coefficient, handle missing data using parwise complete observations

cor_table <- postnorm_postscale_cont_vars %>%

cor(use = "pairwise.complete.obs", method = "spearman")

# use 'correlation' package to filter for variables with spearman's rho >= 0.5. This data will be used to exclude multicolinear variables from LCA

colinear_vars <- postnorm_postscale_cont_vars %>%

correlation(method = "spearman") %>%

filter(rho >= abs(0.5))

colinear_vars

# Set colour scheme for correlation plot

corr_palette <- divergingx_hcl(5, "Spectral")

# Check for missing or infinite values in correlation matrix

any_missing <- any(is.na(cor_table))

any_infinite <- any(!is.finite(cor_table))

# If there are missing or infinite values, you may need to handle them appropriately

if (any_missing || any_infinite) {

# Identify and handle missing or infinite values

# For example, you can replace missing values with 0 or handle them based on your specific requirements

# Replace missing values with 0

cor_table[is.na(cor_table)] <- 0

# Replace infinite values with a large finite value

cor_table[!is.finite(cor_table)] <- 1e6

}

# Proceed with creating the correlation plot

shade_plot <- corrplot(cor_table,

method = "shade",

type = "lower",

col = colorRampPalette(corr_palette)(200),

shade.col = NA,

tl.col = "black",

tl.pos = "l",

addgrid.col = "black",

addCoef.col = "black",

cl.pos = "b",

number.font = 1,

order = "AOE",

diag = FALSE)

# Construct correlation plots for publication

shade_plot <- corrplot(cor_table,

method = "shade",

type = "lower",

col = colorRampPalette(corr_palette)(200),

shade.col = NA,

tl.col = "black",

tl.pos = "l",

addgrid.col = "black",

addCoef.col = "black",

number.font = 2,

cl.pos = "b",

order = "AOE",

diag = FALSE)

# Set the global font family

par(family = "Arial")

# Create the correlation plot

circle_plot <- corrplot(cor_table,

method = "circle",

type = "upper",

col = colorRampPalette(corr_palette)(200),

tl.col = "black",

addgrid.col = "black",

cl.pos = "n",

number.font = 0.1,

order = "AOE",

diag = FALSE,

tl.cex = 0.6) # Adjust the font size

# Display the correlation plot

print(circle_plot)

#Run LCA for 1-5 classes

PoDB_Ready_21July <- cbind(data[, -which(names(data) %in% colnames(postnorm_postscale_cont_vars))], postnorm_postscale_cont_vars)

write.csv(PoDB_Ready_21July, "PoDB_Ready_21July.csv"

# recode NA values to "." for mplus

PoDB_Ready_21July[is.na(PoDB_Ready_21July)] <- "."

#Prepare data for Mplus

prepareMplusData( PoDB_Ready_21July,

filename = "PoDB_Ready_21July.dat",

keepCols = c("PT_ID", "AGE", "SEX", "SURG", "EDU", "DIAB", "HYPERTEN",

"GDS", "BADL", "VVAS", "NART", "LETTER", "CATEG",

"COLOUR2", "NYPR", "ACB", "INTPENT", "ORIENT", "ASA",

"ORIENTCHANGE", "RECALL", "ThreeSC", "INATTEN", "ALTCONC",

"MinSpO2", "MaxTemp", "MinSBP", "MaxHR", "MEq", "IL1b",

"IL6", "IL8", "TNFa", "AB4240", "GFAP",

"NFL", "sTREM2", "PDGFRb","pTau181", "Qalb")

# Create mplus .inp files for 1-5 classes, using "MPlusSyntax.txt" as a template file

createModels("MPlusSyntax.txt")

# Run models

runModels("mplus/PoDB", logFile = "mplus/PoDB/logfile.log")

# Read output

lca <- readModels("mplus/PoDB")

# Construct dtaframe with normalised, centred data used in LCA, with patient id (R format i.e. NA == "NA").

zscores <- cbind(data[, -which(names(data) %in% colnames(postnorm_postscale_cont_vars))], postnorm_postscale_cont_vars)%>%

# Analyse output, constructing summary table, BIC elbow plot, parameter plots for each lca model

# Pass 'zscores' to custom function - used to produce parameter plots

lca_compare <- lca_compare(lca, zscore_df = zscores)

lca_compare

# Exctract class allocations and merge to data

allocations <- map(lca, ~.x$savedata$C) %>%

reduce(cbind)

colnames(allocations) <- str_c(english(1:length(lca)),"_class_alloc")

allocations <- cbind(data, allocations)

```

# 3-Class model has lowest BIC but 2-class model has next lowest BIC and is shown to be better than one class model by T11_VLMR_Pvalue.

# Progress with 2-class model and investigate.

# Entropy ranges from 0-1 with higher values indicating better accuracy of class allocation.

# Descriptive statistics and outcome measures by latent class for 2 class model

# Merge class allocation and probabilites with original dataset

PoDB_lca <- lca$X2.class.out$savedata %>%

select(CPROB1, CPROB2, C, PT_ID) %>%

right_join(data, by = c("PT_ID" = "PT_ID"))

# Identify categorical variables

categorical_vars <- c("SEX", "SURG", "DIAB", "HYPERTEN", "INTPENT", "ASA",

"RECALL", "ThreeSC", "INATTEN", "ALTCONC", "CAM",

"CAM/Rep")

# Convert categorical variables to factors

PoDB_lca[categorical_vars] <- lapply(PoDB_lca[categorical_vars], factor)

# Check the structure of the data frame

str(PoDB_lca)

# save csv

write_csv(PoDB_lca, "PoDB_lca.csv")

# use gtsummary package to create a table of descriptive stats stratified by LCA class

comparison_table <- PoDB_lca %>%

select("AGE", "SEX", "SURG", "EDU", "DIAB", "HYPERTEN",

"GDS", "BADL", "VVAS", "NART", "LETTER", "CATEG",

"COLOUR2", "NYPR", "ACB", "INTPENT", "ORIENT", "ASA",

"ORIENTCHANGE", "RECALL", "ThreeSC", "INATTEN", "ALTCONC",

"MinSpO2", "MaxTemp", "MinSBP", "MaxHR", "MEq", "IL1b",

"IL6", "IL8", "TNFa", "AB4240", "GFAP",

"NFL", "sTREM2", "PDGFRb", "pTau181", "Qalb", C) %>%

mutate(subphenotype = fct_relevel(fct_recode(as.character(C),

"Subphenotype 1" = "1",

"Subphenotype 2" = "2"),

"Subphenotype 1",

"Subphenotype 2"),

.keep = "unused") %>%

tbl_summary(by = subphenotype,

missing = "no",

label = list("AGE" ~ "Age (years)",

"SEX" ~ "Sex",

"SURG" ~ "Surgery Type (Hip or Knee)",

"EDU" ~ "Years in education",

"DIAB" ~ "Diabetes yes/no",

"HYPERTEN" ~ "Hypertension yes/no",

"GDS" ~ "GDS (preop)",

"BADL" ~ "BADL (preop)",

"VVAS" ~ "VVAS at rest (preop)",

"NART" ~ "NART Score (preop)",

"LETTER" ~ "Letter fluency (preop)",

"CATEG" ~ "Category fluency (preop)",

"COLOUR2" ~ "Colour trails 2 time (preop)",

"NYPR" ~ "NYPR delayed recall (preop)",

"ACB" ~ "ACB",

"INTPENT" ~ "Intersecting Pentagons (preop)",

"ORIENT" ~ "Orientation score (preop)",

"ASA" ~ "ASA status",

"ORIENTCHANGE" ~ "Orientation periop change",

"RECALL" ~ "Three object recall (postop)",

"ThreeSC" ~ "Three step command (postop)",

"INATTEN" ~ "Inattention (postop)",

"ALTCONC" ~ "Altered level of consciousness (postop)",

"MinSpO2" ~ "Minimum SpO2 (postop)",

"MaxTemp" ~ "Maximum Temperature (postop)",

"MinSBP" ~ "Minimum systolic blood pressure (postop)",

"MaxHR" ~ "Maximum heart rate (postop)",

"MEq" ~ "Total morphine equivalents (postop)",

"IL1b" ~ "IL1b periop change",

"IL6" ~ "IL6 periop change",

"IL8" ~ "IL8 periop change",

"TNFa" ~ "TNFa periop change",

"AB4240" ~ "AB4240 ratio",

"GFAP" ~ "CSF GFAP (preop)",

"NFL" ~ "CSF NFL (preop)",

"sTREM2" ~ "CSF sTREM2 (preop)",

"PDGFRb" ~ "CSF PDGFRb (preop)",

"pTau181" ~ "Plasma ptau181 (preop)",

"Qalb" ~ "Serum CSF Albumin ratio (preop)")) %>%

add_p(test = all_categorical() ~ "fisher.test",

test.args = all_tests("fisher.test") ~ list(simulate.p.value = TRUE)) %>%

add_q(method = "bonferroni") %>%

bold_labels()

comparison_table

# Set the figure width and height (adjust these values as needed)

options(

repr.plot.width = 20, # Width in inches

repr.plot.height = 30 # Height in inches

)

# create comparsion boxplots for each descriptive variable, split by subphenotype

bplots_by_class <- PoDB_lca %>%

select(c(where(is.numeric), -c(CPROB1, CPROB2, PT_ID))) %>%

mutate(subphenotype = fct_relevel(fct_recode(as.character(C),

"Subphenotype 1" = "1",

"Subphenotype 2" = "2"),

"Subphenotype 1",

"Subphenotype 2"),

.keep = "unused") %>%

pivot_longer(cols = !matches("^subphenotype$"), names_to = "variable", values_to = "value") %>%

ggplot(aes(x = subphenotype, y = value)) +

geom_boxplot(aes(fill = subphenotype)) +

facet_wrap(~variable, ncol = 5, nrow = 6, scale = "free", strip.position = "right") +

theme(strip.text = element_text(margin = margin(b = 10, t=10)),

strip.background = element_blank()) +

theme_classic() +

theme(axis.title.x = element_blank(),

axis.text.x = element_blank(),

axis.ticks.x = element_blank(),

strip.text = element_text(size = 6),

legend.position = "none") +

scale_color_discrete_diverging(palette = "Blue-Red 2", nmax = 4, order = 2:3, aesthetics = "fill")

bplots_by_class

#Organising and visuallising the categorical variables.

# Select patient ID, class assignment, and categorical variables

Categ_Vars <- PoDB_lca %>%

select(PT_ID, C, SEX, SURG, DIAB, HYPERTEN, INTPENT, ASA,

RECALL, ThreeSC, INATTEN, ALTCONC, CAM, `CAM/Rep`)

colnames(Categ_Vars)[colnames(Categ_Vars) == "C"] <- "Class Allocation"

print(Categ_Vars)

class_split <- split(Categ_Vars, Categ_Vars$"Class Allocation")

class_one_data <- class_split$`1`

class_two_data <- class_split$`2`

# Printing the first few rows of each dataframe

print(head(class_one_data))

print(head(class_two_data))

# List of categorical variable names

categorical_vars <- c("SEX", "SURG", "DIAB", "HYPERTEN", "INTPENT", "ASA",

"RECALL", "ThreeSC", "INATTEN", "ALTCONC", "CAM",

"CAM/Rep")

# Function to calculate proportions and return a named vector

calculate_proportions <- function(data) {

prop.table(table(data)) * 100

}

# Calculate proportions for class one data

proportions_class_one <- lapply(class_one_data[categorical_vars], calculate_proportions)

# Calculate proportions for class two data

proportions_class_two <- lapply(class_two_data[categorical_vars], calculate_proportions)

# Print proportions for class one

print(proportions_class_one)

# Print proportions for class two

print(proportions_class_two)

# List of categorical variable names and their corresponding labels

categorical_vars <- c("SEX", "SURG", "DIAB", "HYPERTEN", "INTPENT", "ASA",

"RECALL", "ThreeSC", "INATTEN", "ALTCONC", "CAM",

"CAM/Rep")

# Function to calculate proportions and return a data frame

calculate_proportions <- function(data, variable_name, class) {

prop <- prop.table(table(data)) * 100

data.frame(Variable = variable_name, Category = names(prop), Proportion = prop, Class = class)

}

# Calculate and store proportions for each variable in both classes

proportions_list <- lapply(categorical_vars, function(var) {

class_one_proportions <- calculate_proportions(class_one_data[[var]], var, "1")

class_two_proportions <- calculate_proportions(class_two_data[[var]], var, "2")

rbind(class_one_proportions, class_two_proportions)

})

# Combine the list of proportions data frames into a single data frame

merged_proportions <- do.call(rbind, proportions_list)

# Print the merged proportions

print(merged_proportions)

```

# z-score line plot for publication

# Assuming lca$X2.class.out$savedata is your dataframe with raw data

# Assuming zscores is your dataframe with zscores

# Assuming the rest of your code remains the same

# Create a vector of variable names to exclude from the x-axis

variables_to_exclude <- c("SEX", "SURG", "DIAB", "HYPERTEN", "INTPENT", "ASA",

"RECALL", "ThreeSC", "INATTEN", "ALTCONC")

# Filter the dataframe to exclude the specified variables

zscores_by_var_subphenotype_ <- zscores_by_var_subphenotype_ %>%

filter(!variable %in% variables_to_exclude)

# Make a dummy dataframe used to relevel factor zscores_by_var_subphenotype$variable

relevel_df <- zscores_by_var_subphenotype_ %>%

filter(subphenotype == "Subphenotype 1") %>%

mutate(variable = fct_reorder(variable, mean_zscore))

zline <- zscores_by_var_subphenotype_ %>%

mutate(variable = factor(variable, levels = levels(relevel_df$variable))) %>%

ggplot(aes(x = variable, y = mean_zscore, group = subphenotype)) +

geom_line(aes(colour = subphenotype)) +

ylab("Mean z-score") +

xlab("Continuous Variable") +

ggtitle("Determinant continuous variables in LCA by subphenotype") +

theme_classic() +

theme(legend.title = element_blank(),

axis.text.x = element_text(angle = 90, vjust = 0.5, hjust = 1, size = 10),

plot.title = element_text(hjust = 0.5, size = 10),

axis.title.x = element_blank(),

legend.position = c(0.2, 0.9)) +

scale_colour_manual(values = diverging_hcl(2, "Blue-Red 2"))

zline

print(zline)

#Continuous variable heatmap

# Pivot the data to create a matrix

z_scores_matrix <- zscores_by_var_subphenotype_ %>%

pivot_wider(names_from = subphenotype, values_from = mean_zscore, values_fill = NA) %>%

column_to_rownames(var = 'variable')

# Convert the resulting data frame into a matrix

z_scores_matrix <- as.matrix(z_scores_matrix)

# Rows represent variables and columns represent latent classes

# Set up color palette

my_palette <- colorRampPalette(c("blue", "white", "red"))(n = 100)

# Adjust margins

par(mar = c(5, 4, 4, 4) + 0.1)

# Create the heatmap

heatmap(z_scores_matrix,

cex.axis = 1, # Adjust this value to change the font size

cex.lab = 0.5, # Adjust this value to change the x-axis label font size

cexCol = 0.8,

Colv = NA,

Rowv = NA,

col = my_palette,

scale = "none",

margins = c(8, 8),

main = "Z-Score Heatmap",

xlab = "Latent Classes",

ylab = "Variables")

# Add color key (legend)

legend("bottomright",

legend = c("Low", "Medium", "High"),

fill = colorRampPalette(c("blue", "white", "red"))(3),

title = "Z-Scores")

data("allocations")

write.table(allocations, file = "allocations.txt", sep = "\t",

row.names = TRUE, col.names = NA)

write.csv(allocations, file = "allocations.csv")

write_csv2(allocations, file = "allocations.csv")

PoDB_Ready_21July <- read.table("PoDB_Ready_21July.dat", header = TRUE)

write.csv(PoDB_Ready_21July, "PoDB_Ready_21July.csv", col.names = TRUE)

# Using base R

colnames(PoDB_lca)[colnames(PoDB_lca) == "C"] <- "Class Allocations"

library(stats)

class_1_data <- PoDB_lca[PoDB_lca$"Class Allocations" == 1, ]

class_2_data <- PoDB_lca[PoDB_lca$"Class Allocations" == 2, ]

variables_to_remove <- c("CPROB1", "CPROB2", "PT_ID") # Replace with actual variable names

class_1_data <- class_1_data[, !(names(class_1_data) %in% variables_to_remove)]

class_2_data <- class_2_data[, !(names(class_2_data) %in% variables_to_remove)]

# Create the barcharts with altered fill colors

p <- ggplot(merged_proportions, aes(x = Category, y = Proportion.Freq, fill = factor(Class))) +

geom_bar(stat = "identity", position = "dodge") +

facet_wrap(vars(Variable), scales = "free", ncol = 4) +

labs(x = "Category", y = "Proportion", fill = "Class") +

scale_fill_manual(values = c("deepskyblue", "indianred1")) + # Set custom colors for Class 1 and Class 2

theme_bw()

print(p)

```

1. **Mplus Syntax**

[[init]]

iterators = classes;

classes = 1:5;

filename = "[[classes]]-class.inp";

outputDirectory = "mplus/PoDB/";

[[/init]]

Title: [[classes]]-class LCA of PoDB Data;

Data: File is "/Users/emilybowman/PoDBLCA/21July/PoDB_Ready_21July.dat";

Variable:

Names are PT_ID AGE SEX SURG EDU DIAB HYPERTEN GDS BADL VVAS NART LETTER CATEG COLOUR2 NYPR ACB INTPENT ORIENT ASA ORIENTCHANGE RECALL ThreeSC INATTEN ALTCONC MinSpO2 MaxTemp MinSBP MaxHR MEq IL1b IL6 IL8 TNFa AB4240 GFAP NFL sTREM2 PDGFRb pTau181 Qalb;

CATEGORICAL are SEX SURG DIAB HYPERTEN INTPENT ASA RECALL ThreeSC INATTEN ALTCONC;

Missing are .;

Idvariable is PT_ID;

usevar AGE-Qalb;

CLASSES = c ([[classes]]);

ANALYSIS:

Processors = 4;

TYPE = MIXTURE;

starts = 200 50;

OUTPUT: sampstat residual tech11 tech14;

PLOT: type is plot3;

SAVEDATA:

FILE = "[[classes]]_class_corr.txt";

SAVE = CPROBABILITIES;
