# Supplementary material for: Identifying subphenotypes of patients undergoing post‐operative delirium assessment
Source: Alzheimers Dement. 2025 Jul 16;21(7):e70516. doi: 10.1002/alz.70516 (PMC12265012; doi:10.1002/alz.70516)
Supplement: Supplementary file 3 — Appendix Table A [file ALZ-21-e70516-s007.docx]

**Appendix Table A: Pooled Inter-Plate %CV for plasma and CSF analyses.**

|  | **Internal Control Type** | **Marker** | **Inter-Plate %CV** |
| --- | --- | --- | --- |
| **MSD Proinflammatory Panel 1** | Pooled plasma | IL-1β | 25.54 |
|  | Pooled plasma | IL-6 | 29.21 |
|  | Pooled plasma | IL-8 | 25.46 |
|  | Pooled plasma | TNF-α | 29.47 |
|  | Pooled CSF | IL-1β | 12.88 |
|  | Pooled CSF | IL-6 | 2.93 |
|  | Pooled CSF | IL-8 | 6.14 |
|  | Pooled CSF | TNF-α | 21.26 |
| **Quanterix 4plexE Assay** | Pooled plasma | AB40 | 4.52 |
|  | Pooled plasma | AB42 | 3.80 |
|  | Pooled plasma | GFAP | 5.69 |
|  | Pooled plasma | NFL | 3.03 |
|  | Pooled CSF | AB40 | 10.85 |
|  | Pooled CSF | AB42 | 35.67 |
|  | Pooled CSF | GFAP | 5.92 |
|  | Pooled CSF | NFL | 8.35 |
|  | Pooled plasma | pTau-181 | 10.65 |
|  | Pooled CSF | TREM2 | 12.99 |
