# Supplementary material for: Identifying subphenotypes of patients undergoing post‐operative delirium assessment
Source: Alzheimers Dement. 2025 Jul 16;21(7):e70516. doi: 10.1002/alz.70516 (PMC12265012; doi:10.1002/alz.70516)
Supplement: Supplementary file 4 — Appendix Table B Appendix Section C – Description of correlation analysis. The Correlation Matrix was calculated using the Spearman method. These correlations are also shown in the dot plot. The post‐normalisation density plots are shown below. [file ALZ-21-e70516-s006.docx]

**Appendix Table B**

A list of potential indicators for the PoDB LCA and their abbreviated names.

| **Full Variable Name** | **Abbreviation** |
| --- | --- |
| Age | AGE |
| Sex | SEX |
| Surgery type | SURG |
| Years in education | EDU |
| Diabetes | DIAB |
| Hypertension | HYPERTEN |
| Geriatric Depression Score | GDS |
| Visuo Vertigo Analogue Score of Pain at rest | VVAS |
| National Adults Reading Test score | NART |
| Letter fluency | LETTER |
| Category fluency | CATEG |
| Stroop test | STROOP |
| Colour trails 2 | COLOUR2 |
| New York Paragraph delayed recall test | NYPR |
| Anticholinergic Burden | ACB |
| Intersecting Pentagons | INTPENT |
| Orientation | ORIENT |
| American Society of Anaesthesiologists Physical status | ASA |
| Preoperative plasma pTau181 | pTau181 |
| Preoperative serum albumin. | Qalb |
| CSF AB4240 | AB4240 |
| CSF GFAP, | GFAP |
| CSF NfL, | NFL |
| CSF sTREM2, | sTREM2 |
| CSF PDGFRb. | PDGFRb |
| Perioperative change in Orientation | ORIENTCHANGE |
| Perioperative change in plasma IL-1Β | IL1b |
| Perioperative change in plasma IL-6 | IL6 |
| Perioperative change in plasma IL-8 | IL8 |
| Perioperative change in plasma TNF-α | TNFa |
| Postoperative day 1 Three object recall | RECALL |
| Postoperative day 1 Three step command | ThreeSC |
| Postoperative day 1 inattention | INATTEN |
| Postoperative day 1 altered level of consciousness | ALTCONC |
| Postoperative day 1 maximum temperature | MaxTemp |
| Postoperative day 1 minimum Sp02 | MinSpO2 |
| Postoperative day 1 minimum SBP | MinSBP |
| Postoperative day 1 minimum DBP | MinDBP |
| Postoperative day 1 maximum heartrate | MaxHR |
| Total number of morphine equivalents | Meq |
