# Supplementary material for: Identifying subphenotypes of patients undergoing post‐operative delirium assessment
Source: Alzheimers Dement. 2025 Jul 16;21(7):e70516. doi: 10.1002/alz.70516 (PMC12265012; doi:10.1002/alz.70516)
Supplement: Supplementary file 5 — Appendix Table C1 [file ALZ-21-e70516-s002.docx]

**Appendix Table C1-** Results of correlation analysis

| **Parameter 1** | **Parameter 2** | **Spearman’s Rank Correlation Coefficient (rho)** | **95% CI** | **p** |
| --- | --- | --- | --- | --- |
| STROOP | CATEG | 0.55 | [0.46, 0.62] | < .001*** |
| MinDBP | MinSBP | 0.56 | [0.46, 0.65] | < .001*** |
| NART | LETTER | 0.55 | [0.46, 0.62] | < .001*** |
| LETTER | CATEG | 0.57 | [0.48, 0.64] | < .001*** |
| IL-6 | IL-8 | 0.65 | [0.57, 0.72] | < .001*** |
| GFAP | NfL | 0.70 | [0.63, 0.76] | < .001*** |

Table C1: Statistically significant (P<0.05) results of Spearman’s Rank Correlation Analyses between candidate variables for inclusion in the model.

*STROOP = Stroop test, CATEG = Category fluency, MinDBP = Postoperative day 1 minimum Diastolic Blood Pressure, MinSBP = Postoperative day 1 minimum Systolic Blood Pressure, NART = National Adults Reading Test score, LETTER = Letter fluency, IL-6 = Perioperative change in plasma Interleukin-6, IL-8 = Perioperative change in plasma Interleukin-8, GFAP = CSF Glial Fibrillary Acidic Protein, NfL = CSF Neurofilament Light*
