# Supplementary material for: Identifying subphenotypes of patients undergoing post‐operative delirium assessment
Source: Alzheimers Dement. 2025 Jul 16;21(7):e70516. doi: 10.1002/alz.70516 (PMC12265012; doi:10.1002/alz.70516)

Appendix Figure C1: Correlation circle plot displaying correlations between indicators for inclusion in the LCA model.


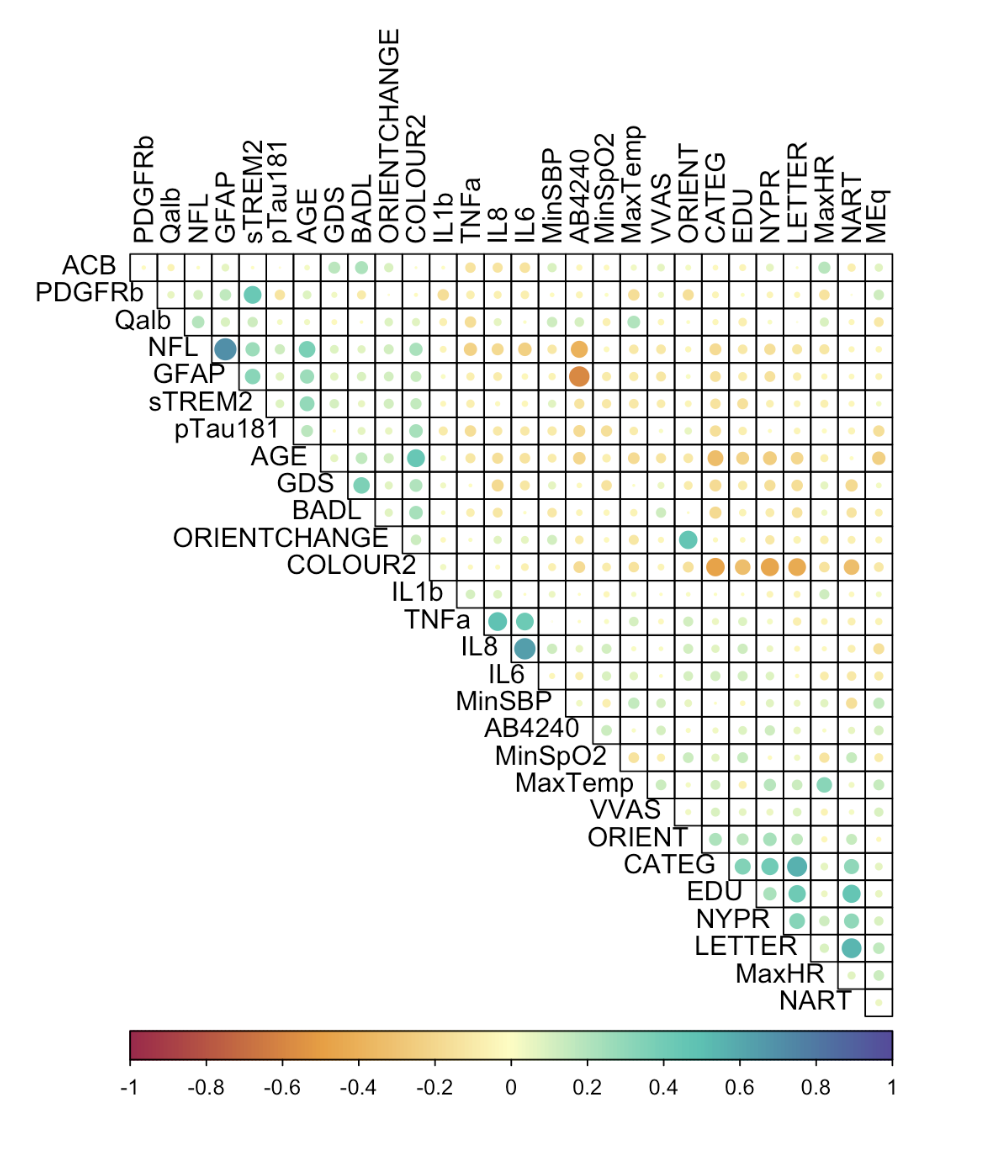

Supplement: Supplementary file 6 — Appendix Figure C1: Correlation circle plot displaying correlations between indicators for inclusion in the LCA model. [file ALZ-21-e70516-s004.docx]
