# Supplementary figures and images for: Identifying subphenotypes of patients undergoing post‐operative delirium assessment
Source: Alzheimers Dement. 2025 Jul 16;21(7):e70516. doi: 10.1002/alz.70516 (PMC12265012; doi:10.1002/alz.70516)

Appendix Figure C2: Post normalisation density plots for the PoDB indicators.


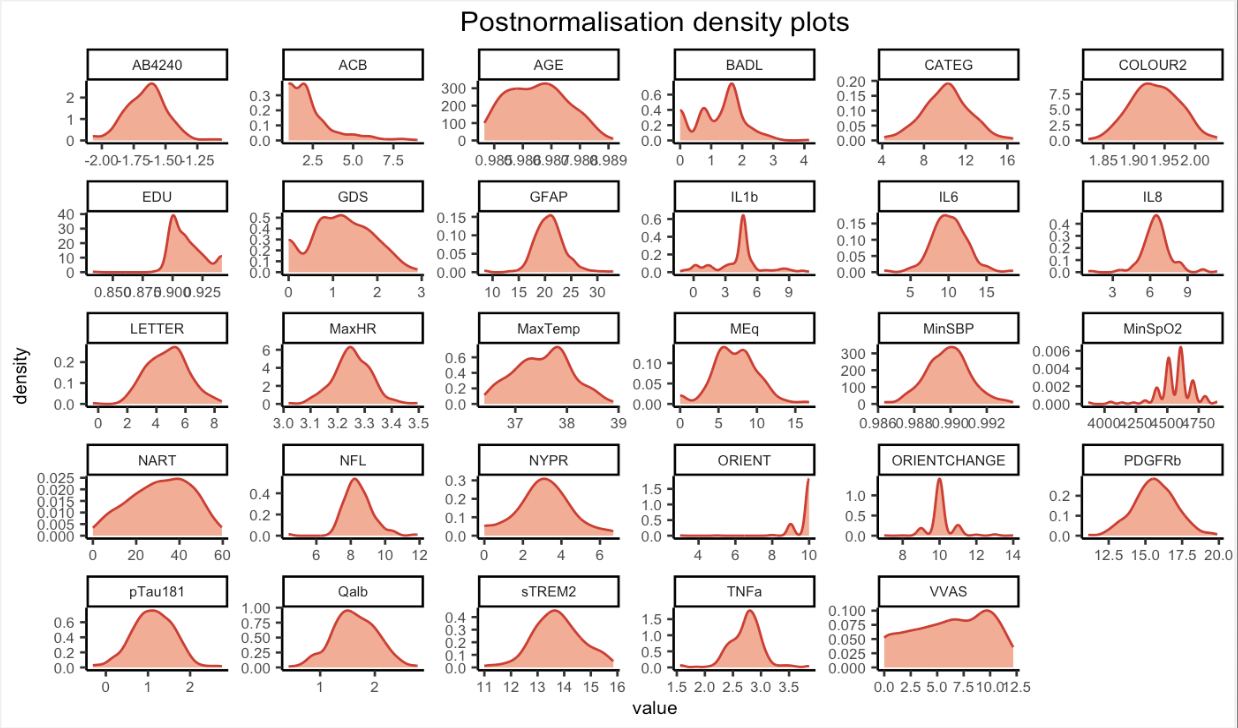

Supplement: Supplementary file 7 — Appendix Figure C2: Post normalisation density plots for the PoDB indicators. [file ALZ-21-e70516-s003.docx]
